# Animals Do Not Induce or Reduce Attentional Blinking, But They Are Reported More Accurately in a Rapid Serial Visual Presentation Task

**DOI:** 10.1177/2041669517735542

**Published:** 2017-10-16

**Authors:** Thomas Hagen, Bruno Laeng

**Affiliations:** 87366 Department of Psychology, University of Oslo, Oslo, Norway

**Keywords:** animacy, animals, attention, evolution, rapid serial visual presentation

## Abstract

Evolutionary psychologists have suggested that modern humans have evolved to automatically direct their attention toward animal stimuli. Although this suggestion has found support in several attentional paradigms, it is not without controversy. Recently, a study employing methods customary to studying the attentional blink has shown inconclusive support for the prioritization of animals in attention. This showed an advantage for reporting animals as second targets within the typical window of the attentional blink, but it remained unclear whether this advantage was really due to a reduction of the attentional blink. We reassessed for the presence of a reduced attentional blink for animals compared with artifacts by using three disparate stimuli sets. A general advantage for animals was found but no indication of a reduction of the attentional blink for animals. There was no support for the prediction that animal distractors should lead to spontaneous inductions of attentional blinks when presented as critical distractors before single targets. Another experiment with single targets still showed that animals were reported more accurately than artifacts. A final experiment showed that when animals were first target, they did not generate stronger attentional blinks. In summary, we did find a general advantage for animal images in the rapid serial visual presentation task, but animal images did not either induce or reduce attentional blinks. This set of results is in line with conclusions from previous research showing no evidence for a special role of animals in attention.

## Introduction

Humans have the remarkable ability to detect and report targets embedded in a stream of rapidly presented images (e.g., 10 images/s; Dux & Marois, 2009). This ability is, however, profoundly reduced when two targets are shown in quick succession. More specifically, if the first target (T1) in a rapid serial visual presentation task (RSVP) reaches awareness (i.e., it gets reported explicitly), then a second target (T2) presented within a short subsequent period of time of about 500 ms is suppressed or unable to reach awareness. As the effect seems to not only be sensitive to a temporal window but also to the number of intervening items between T1 and T2 (Dux & Marois, 2009), the number of stimuli presented (at a rate of about 100 ms) from the onset of T1 to the onset of T2 or temporal “lag” is typically manipulated in such studies. Importantly, the decrement in performance does not seem to stem purely from a limit in sensory processing, as the difficulty in detecting T2 appears to depend on the requirement of also reporting T1. In cognitive terms, when attentional resources have been employed to process T1, an attentional gate is closed for further processing until the processing of the selected item has completed (Chun & Potter, 1995; Raymond, Shapiro, & Arnell, 1992). As it appears to depend on the ability of attention to select an item for further processing, this deficit is referred to as an attentional “blink,” as a temporary blindness for any visual input.

Human ancestors most likely lived in an environment where they were faced with cluttered and complex visual settings while being required to behave adaptively. Thus, selective attention, the process of enhancing behaviorally relevant inputs, should have been of central importance. Indeed, being able to react rapidly in relation to competing organisms should have clear benefits for the natural selection of the organism. Consequently, if attention is currently engaged, it should have provided adaptive value if stimuli relevant for survival were able to redirect attention and gain prioritized access to awareness, such that adaptive behavior could be executed earlier than it otherwise would have been. Thus, an attentional bias, which rapidly selects information relevant for survival, such as food, threat, or significant social cues, should have had fundamental survival value.

**Figure 1.**
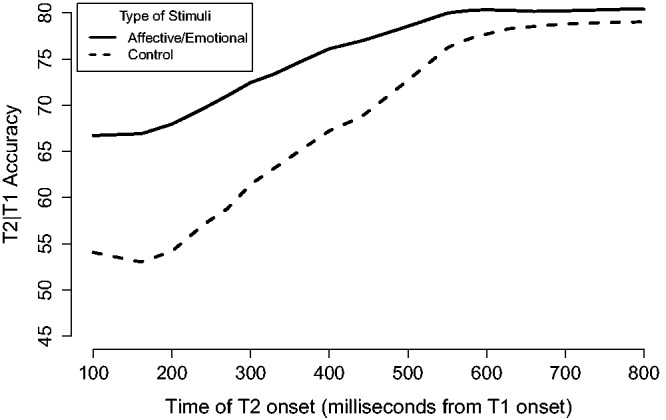
An analysis of T2 reporting accuracy given that T1 was correct (T2*|*T1) for affective or emotional and control stimuli from 10 experiments across six studies (Anderson, 2005; de Oca et al., 2012; Fox et al., 2005; Milders et al., 2006; Ogawa & Suzuki, 2004; Stein, Peelen, Funk, & Seidl, 2010). We extracted accuracies from the individual experiment plots and interpolated between time points before averaging. The figure gives a general overview of the time course of stimuli capable of reducing the impact of the attentional blink in the published literature. Presentation rates varied between 83 and 120 ms in the studies.

Interestingly, the RSVP task has been used to reveal attentional biases, as indexed by an attenuation of the attentional blink, with a variety of stimuli, for example, arousing words (Anderson, 2005; Keil & Ihssen, 2004; Ogawa & Suzuki, 2004), emotional faces (De Martino, Kalisch, Rees, & Dolan, 2009; Fox, Russo & Georgiou, 2005; Milders, Sahraie, Logan, & Donnellon, 2006; Stein, Peelen, Funk, & Seidl, 2010), and food (Neimeijer, de Jong, & Roefs, 2013). These findings suggest that biologically relevant stimuli can gain preferential access to subjective awareness when attention is currently occupied by counteracting attentional blinking (see Figure 1 for an overview of the typical effect).

Similarly, the RSVP task have been used to demonstrate inductions of attentional blinks by presenting biologically relevant stimuli as critical distractors before targets (Arnell, Killman, & Fijavz, 2007; Ciesielski, Armstrong, Zald, & Olatunji, 2010; Most, Chun, Widders, & Zald, 2005; Neimeijer et al., 2013). With the result that targets are harder to detect due to the automatic attentional capture of the (task irrelevant) critical distractors.

Recently, Guerrero and Calvillo (2016) reported that “animacy” increases second target reporting in the RSVP task, when the first target is reported correctly (see also Balas & Momsen, 2014; Kanske, Schönfelder, & Wessa, 2013, for similar advantages of animals over plants). More specifically, they found that animate T2 images (animals) were named more accurately than inanimate T2 images (artifacts, man-made objects) at Lags 2 and 4 (corresponding to 200 ms and 400 ms following the onset of the first target). As these are both within the window of the typical attentional blink, these findings raise the question of whether animacy actually reduces the attentional blink or, alternatively, that the animal targets simply tend to be reported more accurately in this task. That is to say, if animals selectively reduce the attentional blink, they are likely to be biased in early attentional processes. In contrast, if animals do not reduce the attentional blink but are reported more accurately regardless of being presented within the attentional blink, this could instead be characterized as a bias in later post-attentive processing stages. Interestingly, a reduction of the attentional blink by animals should be expected by the “animate monitoring hypothesis” (New, Cosmides, & Tooby, 2007), which states that modern humans have evolved to preferentially and automatically allocate attention toward animals. A similar proposal was offered by Laws (2000), who suggested, based on studies of object naming, that human brains have evolved a processing advantage for living things because of their survival value to humans (see also Crouzet, Joubert, Thorpe, & Fabre-Thorpe, 2012). Thus, separating an effect of naming, or perhaps more likely the speed of perceptual processing, from that of attention seems imperative.

The proposal of an attentional bias for animals is interesting to consider in relation to the literature which shows abundant evidence for the differential processing of animals and non-animals in the human brain (Capitani, Laiacona, Mahon, & Caramazza, 2003; Kriegeskorte et al., 2008; Laws & Neve, 1999; Martin, Wiggs, Ungerleider, & Haxby, 1996; Sha et al., 2015). This has become especially clear from studies of neurological patients indicating that there exists a “double dissociation” in the ability to name or provide semantic knowledge about animals and artifacts (e.g., Hillis & Caramazza, 1991). While it is debated whether these distinctions arise from experience or are imposed by evolution (Laws, 2000; Mahon, Anzellotti, Schwarzbach, Zampini, & Caramazza, 2009), this line of observations suggests that a neural architecture or specialized substrate for the differential processing of animals and non-animals can exist in the human brain (Caramazza & Shelton, 1998; Mahon & Caramazza, 2009, 2011). As a capability of distinguishing between animal and nonanimal things should be a requirement for a circuit capable of rapidly biasing attention toward animals, these observations are interesting in that it seems plausible that evolutionary pressures could have utilized this distinction or implemented parallel circuits based on similar principles.

More specifically, the animate monitoring hypothesis (New et al., 2007) proposes that humans have evolved to automatically deploy attentional resources toward animals regardless of their relevance to the task. To test this, they used a change detection task where animals, artifacts, and plants would rapidly appear and disappear within a scene. The main finding was that, when the changing object happened to be an animal, the changes were noticed more readily than with other categories (regardless of size, interest, etc.). However, Hagen and Laeng (2016) showed that the observed advantage, although replicable, was likely to stem from uncontrolled variables pertaining to the particular scene images that were used. Nevertheless, other lines of research have found support for the hypothesis in visual search tasks (Jackson & Calvillo, 2013), inattentional blindness (Calvillo & Hawkins, 2016; Calvillo & Jackson, 2014), and, though inconclusively, with the attentional blink (Guerrero & Calvillo, 2016). Thus, there appears to be suggestions for a prioritization of animals in some attentional paradigms.

According to the proposal by New et al. (2007), animal stimuli should be able to recruit attention regardless of the ongoing task or instructions. We reasoned that, given that such a mechanism is posited to work via an interrupt circuit to ongoing voluntary attention, then the attentional blink paradigm would seem especially suitable to investigate such an account. Especially if a hard-wired bias for detecting animals exists in the human brain, it would seem important to begin identifying under which conditions and on which characteristics of the input it operates. A starting point is to examine the “time course” of the attentional blink as an index of available attentional resources for awareness since it appears to be sensitive to certain classes of prioritized stimuli. Specifically, we expected that if images of animals have a priority in being allocated attentional recourses, then the attentional blink paradigm should be sensitive to such a prioritization. Thus, in the present study, we assessed whether images of animals are (a) reported more successfully in a RSVP task and (b) if they can also reduce or induce attentional blinking. The former possibility, if observed alone, would suggest a postattentional or perceptual advantage for animals, while a combination with the latter should be indicative of an early attentional bias.

Interestingly, the advantage for animate T2 images observed by Guerrero and Calvillo (2016) appeared to be smallest at the 200 ms lag and greatest at the 400 ms lag. Typically, in studies reporting a reduction of the attentional blink, the pattern is the reverse: namely, that short lags lead to more pronounced differences to control stimuli while later lags shows reduced differences (see Figure 1), indicating the stimuli’s specificity to the attentional blink. The peculiarity of these findings may give a hint that the animacy advantage previously observed may not actually be due to a modulatory effect on the attentional blink per se.

## Experiment 1

In Experiment 1A, we set out to replicate the original findings of Guerrero and Calvillo (2016) by using the same set of images, as referenced in their article. Although the original study investigated threat in addition to animacy, we collapsed the threat conditions into just two main categories: animals (originally: animate threatening and non-threatening) and artifacts (originally: inanimate threatening and non-threatening).

As Guerrero and Calvillo (2016) noted that they did not attempt to control for any low-level characteristics of the stimuli, it was deemed important to investigate if the advantage for animals could also be observed with a different set of images which were pre-experimentally balanced on low-level characteristics. Thus, in Experiment 1B, we used the set of images from Experiment 1 in Hagen and Laeng (2016), which had been balanced on low-level visual characteristics (contrast, size, saliency, and saturation) and had, as a matter of fact, resulted in no advantages for animals in a change detection task. From our estimates, the original study showed a minor increase in accuracy of 8.2% for animals at Lag 2 (i.e., onset at 200 ms after T1 onset) and a more substantial advantage of 17.1% at Lag 4 (i.e., 400 ms after T1 onset). Thus, it appears that if animals are effective in reducing the attentional blink, this is most effective at late rather than early lags. Furthermore, if the advantage is really specific to and caused by the attentional blink, then we would also expect a significant reduction of the advantage for animals outside the typical window of the attentional blink at Lag 7 (700 ms after T1 onset), if it follows the typically pattern in similar studies (see Figure 1). Thus, we predicted an interaction where animals should be reported more accurately than artifacts at Lag 4 and not reported more accurately than artifacts at Lag 7. Conversely, the null hypothesis was that we observe only main effects of category and lag.

Guerrero and Calvillo’s study required participants to name the objects, but this requirement seems unnecessary in relation to the animate monitoring hypothesis and perhaps problematic. The literature describes a normal tendency for category-specific effects in naming; for example, Laws and Neve (1999) observed an advantage for naming briefly presented animal images (see also Laws, 2000). Explicit semantic level processing should not be necessary for the automatic attentional capture by animals (New et al., 2007). Thus, we chose to use a visual array with distractors and targets, where participants indicated each target by clicking on the corresponding image. While this cannot rule out semantic level processing, we expected that this response would keep minimal the influence of factors pertaining to naming (e.g., lexical retrieval) the objects explicitly.

Since Guerrero and Calvillo (2016) also observed that when animals were T2, the T1 accuracy was higher, we planned to assess this aspect as well. A major difference with their study is that in Experiment 1B we used a set of balanced images (Hagen & Laeng, 2016) in the attempt to control for a set of potential confounds (contrast, size, saliency, and saturation). In summary, despite some changes, we expected to replicate the original findings and observe a reduction of the advantage for animals at Lag 7 as compared with Lag 4.

Power analysis assuming a population standard deviation of 10, a correlation between repeated measures of 0.3, and an advantage for animals of 17.1% at Lag 4 and 0% at Lag 7 showed that we would need at least 10 participants to have a power of 80% in detecting a significant interaction. However, to encompass a potentially 10% smaller advantage at Lag 4 (which could be expected from Figure 1) or a 10% residual advantage at Lag 7, we aimed to test approximately 50 participants.

### Methods

#### Participants

We recruited 59 participants for Experiment 1A (15 females) with a mean age of 30.8 years (range: 18–55 years, *SD*: 8.63 years) and 54 participants for Experiment 1B (17 females) with a mean age of 34.2 years (range: 21–64 years, *SD*: 9.1 years). All were recruited with *Crowdflower*® and randomly assigned to either experiment. All participants agreed to an informed consent approved by the institute’s internal review board (ref.: 888026) and in accordance with the Declaration of Helsinki.

#### Apparatus

The experiment was implemented with JavaScript and each participant ran the experiment on their own computer (see Crump, McDonnell, & Gureckis, 2013; Semmelmann & Weigelt, 2016 for similar RSVP implementations).

#### Stimuli

For Experiment 1A, we used the same set of photographs of objects on a white background as in Guerrero and Calvillo (2016). For Experiment 1B, we used the same stimuli as in Experiment 1A, except for a different set of T2 target images balanced on low-level variables (i.e., the same set of photographs used for Experiment 1 in Hagen & Laeng, 2016).

#### Procedure

**Figure 2.**
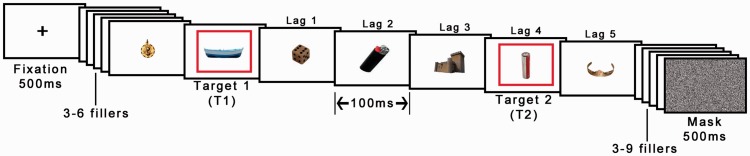
Example of a RSVP sequence (time runs from left to right). Each image was displayed for 100 ms. Target objects displayed in red frames. Lag were manipulated by either displaying the second red frame after three (Lag 4) or six (Lag 7) intervening images. The words beneath and above the images were not shown in the actual experiment. Each image was displayed for 100 ms, for example, Lag 4 onset corresponds to 400 ms from T1 onset (© 2012 Moreno-Martínez, Montoro, PLoS One, 7(5), p. e37528.).

**Figure 3.**
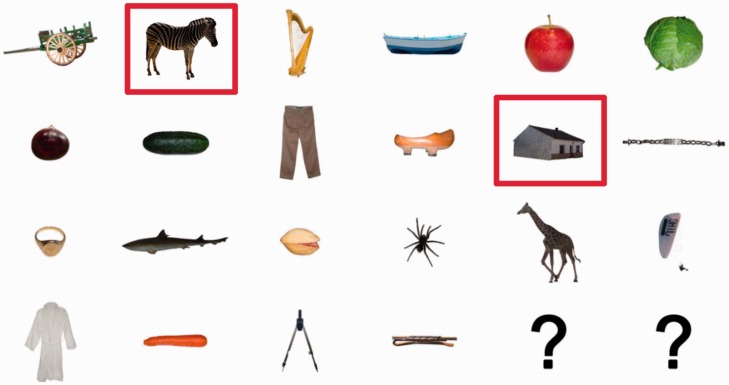
Example of the response array. Participants were instructed to select the first target and then the second target. Red frames appeared around the objects as participants selected them. If they did not remember a target, they selected one of the question marks (© 2012 Moreno-Martínez, Montoro, PLoS One, 7(5), p. e37528.).

Each trial started with the presentation of a fixation cross in the center of the display for 500 ms. Then, a series of 18 images were presented at a rate of 100 ms each, two of which were indicated as targets by a red frame (see Figure 2). The first target (T1) was presented at one of the following positions in the stream: fourth, fifth, sixth, or seventh. The second target (T2) was presented at either Lag 4 (400 ms after T1 onset) or Lag 7 (700 ms after T1 onset) after T1, corresponding to three and six intervening images between T1 and T2. T1 images belonged to four distinct categories (fruit, vegetables, musical instruments, or furniture), whereas T2 images were either animals or artifacts. Crucially, T1 positions and T1 categories were both counterbalanced across T2 categories and lags. Distractor images were randomly chosen from a pool of 103 images (none of which was of an animal). The experiment consisted of 80 trials (two blocks of 40 trials); thus, each T2 image were shown twice but randomly paired with a different T1 category image. After the stream of images completed, a noise mask was presented for 500 ms and, subsequently, a visual array of 22 objects and 2 question marks were presented (see Figure 3). The array contained a random selection of 13 distractor objects from the stream as well as the two targets and seven T1 and T2 objects. Participants selected the two target objects in the order that they saw them or, if they did not remember, they selected a question mark. Before starting the experiment, participants were required to complete five practice trials where the first two had a presentation rate of 400 and 200 ms, respectively. Participants were only allowed to continue to the main experiment after correctly reporting all targets in at least three of the five practice trials.

### Results

#### Experiment 1A

**Figure 4.**
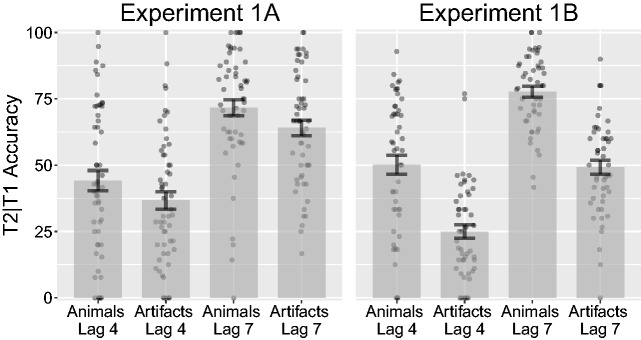
Combined bar and scatter plots on second target (T2) accuracy when the first target (T1) was correct (T2*|*T1), from Experiments 1A and 1B. Error bars show standard error and the superimposed scatterplots show mean values from each participant. Experiment 1A used the same stimuli as in Guerrero and Calvillo (2016), while Experiment 1B used T2 objects from Hagen and Laeng (2016). Lags 4 and 7 correspond to 400 and 700 ms after T1 onset.

Before conducting the analysis, we removed four participants for having mean T1 accuracy lower than 40% (1.5 *SD* from the mean, 6.8% of all trials) after inspecting the distribution of responses. An ANOVA on T2 accuracy given that T1 was correctly reported (T2*|*T1) on lag (4, 7) and category (animals, artifacts) revealed significant main effects of lag, *F*(1, 54) = 80.18, *p < *.001, $\eta_{P}^{2}$ = .60, and category, *F*(1, 54) = 23.55, *p < *.001, $\eta_{P}^{2}$ = .30. The interaction was not significant, *F*(1, 54)* < *0.01, *p* = .964, $\eta_{P}^{2}$* < *.01 (see Figure 4). At Lag 4, animals had a mean accuracy of 44.2% (*SD* = 28.2%), while artifacts had a mean accuracy of 36.8% (*SD* = 24.4%). At Lag 7, animals had a mean accuracy of 71.7% (*SD* = 22.3%), while artifacts had a mean accuracy of 64.1% (*SD* = 21.5%). To further investigate the specificity of the advantage to the attention blink, we subtracted T2*|*T1 accuracy for artifact targets from animal targets at both Lags 4 and 7 and directly compared for a change in performance between the two lags. The difference was however not significant, *F*(1, 54)* < *0.01, *p* = .974, $\eta_{P}^{2}$* < *.01. Next, to investigate whether T1 accuracy would depend on the category of T2, we ran an ANOVA on T1 accuracy. There were neither significant main effects of lag, *F*(1, 54) = 0.23, *p* = .631, $\eta_{P}^{2}$* < *.01, or category, *F*(1, 54) = 1.93, *p* = .170, $\eta_{P}^{2}$ = .03, nor interactive effects, *F*(1, 54) = 0.02, *p* = .899, $\eta_{P}^{2}$* < *.01.

#### Experiment 1B

Before conducting the analysis, we removed five participants for having mean T1 accuracy lower than 40% (9.3% of all trials). An ANOVA on lag (4, 7) and category (animals, artifacts) revealed significant main effects for lag, *F*(1, 48) = 102.76, *p < *.001, $\eta_{P}^{2}$ = .68, and category, *F*(1, 48) = 135.09, *p < *.001, $\eta_{P}^{2}$ = .74. The interaction was not significant, *F*(1, 48) = 0.70, *p* = .406, $\eta_{P}^{2}$ = .01 (see Figure 4). At Lag 4, animals had a mean accuracy of 50.2% (*SD* = 25.3%), while artifacts had a mean accuracy of 25% (*SD* = 18.1%). At Lag 7, animals had a mean accuracy of 77.7% (*SD* = 14.3%), while artifacts had a mean accuracy of 49.2% (*SD* = 18.2%). To further assess the specificity of the advantage to the attention blink, we subtracted T2*|*T1 accuracy for artifact targets from animal targets at both Lags 4 and 7 and directly compared for a change in performance between the two lags. The difference was however not significant, *F*(1, 48) = 0.62, *p* = .434, $\eta_{P}^{2}$ = .01 Finally, T1 accuracy did not appear to depend on the category of T2, since an ANOVA on T1 accuracy showed no significant main effects of lag, *F*(1, 48) = 1.27, *p* = .266, $\eta_{P}^{2}$ = .03, or category, *F*(1, 48) = 1.35, *p* = .250, $\eta_{P}^{2}$ = .03, and there was no significant interactive effect, *F*(1, 48) = 0.56, *p* = .459, $\eta_{P}^{2}$ = .01.

### Discussion

We found an advantage for animals both with the original set of images and with a different set of target images, which also replicated the main finding of the original study by Guerrero and Calvillo (2016). Remarkably, by using images controlled on low-level characteristics (in Experiment 1B), instead of reducing or erasing the advantage, we largely increased the effect size. It appears that the task is relatively sensitive to the stimuli used, apart from the category it represents; thus, efforts to control the stimuli or performing replications with different sets seem appropriate.

The experiments clearly indicated the presence of an attentional blink, since we observed the hallmark reduction of performance at shorter lags as compared with longer lags. However, our expectation of a reduced advantage at Lag 7 did not find support in the present data. That is, the present pattern of results indicates that images of animals and artifacts are equally affected when presented within the attentional blink window. Thus, we cannot conclude that the advantage for animals results from a reduction of the attentional blink. This draws attention on a potential confound in this experiment as well as Guerrero and Calvillo’s experiment (2016): Animals were only displayed as targets and never as distractors; thus, participants could have learned that whenever they saw an animal, this would necessarily be a target.

While using a visual array response was able to replicate an advantage for animals in this task, one caveat is that it cannot differentiate an effect of RSVP performance from a potential effect of visual search efficiency between the categories (Jackson & Calvillo, 2013; Levin, Takarae, Miner, & Keil, 2001).

Notably, as there were few animal probes, combined with the fact that animals were never distractors, this could have shaped a situation where participants adopted a strategy favoring the detection of animals. The concern is that a visual search advantage combined with potential strategies could have after all inflated the advantage for animals.

## Experiment 2

The previous experiment had some characteristics that could have biased the results in favor of animals or somehow reduced our ability to find an interaction. Namely: (a) animals were always targets and (b) the response consisted in locating and selecting an item within a visual array, which could have benefited animal reporting (Jackson & Calvillo, 2013; Levin et al., 2001). Thus, in this experiment, we changed the frequency of animal distractors to match the frequency of animal targets (25%). Consequently, merely detecting an animal would not be diagnostic of it being a target. In addition, a single image probe per target position substituted the “visual array” response of the previous experiment. Finally, we tested how generalizable are the observed effects when using two different sets of images, namely photographs and line drawings.

Given that reporting accuracy was sensitive to the stimuli used, regardless of their category, it was deemed relevant to see how well the results could extend to a standardized set of images of animals and artifacts, which is also visually very different from those used previously: the line drawings by Snodgrass and Vanderwart (1980). This set of “abstract” images has been used extensively in research on category-specific effects (Caramazza & Mahon, 2003; Caramazza & Shelton, 1998; Laws & Neve, 1999; Stewart, Parkin, & Hunkin, 1992). We made an effort to control for some potential confounding aspects by balancing the stimuli of line drawings on visual complexity and familiarity ratings from Snodgrass and Vanderwart (1980). In the above-mentioned studies, visual complexity and familiarity are commonly controlled and there are indications that visual short-term memory can be enhanced for less complex items (Alvarez & Cavanagh, 2004). Moreover, target familiarity can influence the attentional blink (Shapiro, Caldwell, & Sorensen, 1997; Jackson & Raymond, 2006).

As in the previous experiment, we expected reports to be more accurate for animals than artifacts and that that such an advantage would be revealed at Lag 4 but not at Lag 7. Finally, as in the previous experiment, we also expected an increase in T1 accuracy depending on the category of T2.

### Methods

#### Participants

We recruited 62 participants for Experiment 2A (12 females) with a mean age of 33.8 years (range: 19–58 years, *SD*: 9.98 years) and 64 participants for Experiment 2B (21 females) with a mean age of 31.4 years (range: 14–67 years, *SD*: 10.2 years). All were recruited in the same way as the previous experiment.

#### Apparatus

Identical to the previous experiment.

#### Stimuli

**Figure 5.**
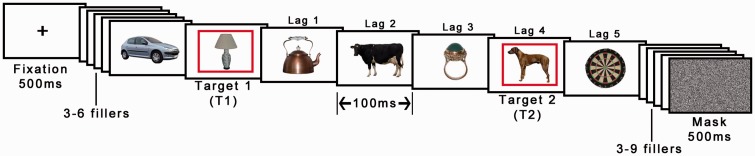
Example of an RSVP sequence in Experiment 2A. Object images © 2012 Moreno-Martínez, Montoro, PLoS One, 7(5), p. e37528.

For Experiment 2A, we used the photographs from as Experiment 1B. For Experiment 2B, we used line drawings (see Figure 5) from the Snodgrass and Vanderwart’s (1980) set. We selected 20 animals (alligator, ant, bird, cat, chicken, cow, dog, donkey, elephant, fox, gorilla, grasshopper, horse, mouse, peacock, pig, rabbit, rooster, snake, and squirrel) and 20 artifacts (airplane, baby carriage, barn, bicycle, cannon, church, french horn, gun, helicopter, iron, motorcycle, rocking chair, roller skate, sailboat, thimble, trumpet, violin, wagon, watch, and whistle). The line drawings of animals and artifacts were matched on size (width, height, number of pixels), familiarity, and visual complexity by selecting pairs of animals and artifacts that were minimally different across measures.

#### Procedure

**Figure 6.**
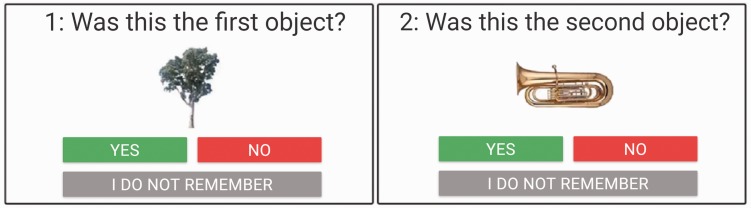
Example of the response screens. Participants were asked to confirm (by pressing “yes”) or deny (by pressing “no”) having seen the objects as targets. Alternately, they could report that they did not remember having seen a target. Object images © 2012 Moreno-Martínez, Montoro, PLoS One, 7(5), p. e37528.

This was identical to Experiment 1, except that we used a single probe image with a three-choice alternative, “yes,” “no,” or “I do not remember” (see Figure 6). Each probe had probability to be correct 50% of the time, by displaying the actual target image. Invalid probe images for T1 objects were randomly chosen from all possible T1 images while invalid T2 probes were categorically congruent with the target 50% of the time. In addition, 25% of the distractor images in the stream were animals (see Figure 5).

### Results

#### Experiment 2A

**Figure 7.**
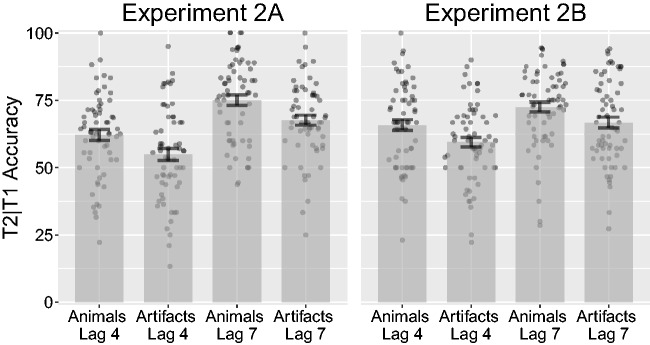
Combined bar and scatter plots on T2*|*T1 accuracy from Experiments 2A and 2B. Error bars show standard error and the superimposed scatterplots show mean values of each participant. Experiment 2A used photographs, while Experiment 2B used line drawings from Snodgrass and Vanderwart (1980).

An ANOVA on T2 accuracy, given that T1 was correct (T2*|*T1), over lag (4, 7) and category (animals, artifacts) revealed a significant main effect of lag, *F*(1, 61) = 47.48, *p < *.001, $\eta_{P}^{2}$ = .44, and category, *F*(1, 61) = 24.92, *p < *.001, $\eta_{P}^{2}$ = .29. The interaction was not significant, *F*(1, 61) = 0.01, *p* = .930, $\eta_{P}^{2}$* < *.01 (see Figure 7). At Lag 4, animals had a mean accuracy of 65.8% (*SD* = 15.7%), while artifacts had a mean accuracy of 59.5% (*SD* = 14.4%). At Lag 7, animals had a mean accuracy of 72.4% (*SD* = 14.4%), while artifacts had a mean accuracy of 66.6% (*SD* = 15.8%). To further investigate the specificity of the advantage to the attention blink, we subtracted T2*|*T1 accuracy for artifact targets from animal targets at both Lags 4 and 7 and directly compared for a change in performance between the two lags. The difference was however not significant, *F*(1, 61) = 0.01, *p* = .937, $\eta_{P}^{2}$* < *.01. An ANOVA on T1 accuracy showed no significant main effects of lag, *F*(1, 61) = 0.18, *p* = .671, $\eta_{P}^{2}$* < *.01, or category, *F*(1, 61) = 0.98, *p* = .325, $\eta_{P}^{2}$ = .02, and the interaction was non-significant, *F*(1, 61) = 0.38, *p* = .542, $\eta_{P}^{2}$* < *.01.

#### Experiment 2B

An ANOVA on T2*|*T1 accuracy over lag (4, 7) and category (animals, artifacts) revealed a significant main effect of lag, *F*(1, 63) = 14.18, *p < *.001, $\eta_{P}^{2}$ = .18, and category, *F*(1, 63) = 16.13, *p < *.001, $\eta_{P}^{2}$ = .20. The interaction was not significant, *F*(1, 63) = 0.02, *p* = .876, $\eta_{P}^{2}$* < *.01 (see Figure 7). At Lag 4, animals had a mean accuracy of 62.1% (*SD* = 15.5%), while artifacts had a mean accuracy of 54.9% (*SD* = 17.3%). At Lag 7, animals had a mean accuracy of 75% (*SD* = 15.5%), while artifacts had a mean accuracy of 67.6% (*SD* = 14.3%). Similarly, when we subtracted T2*|*T1 accuracy for artifact targets from animal targets at both Lags 4 and 7 and directly compared for a change in performance between the two lags, we found no significant effect, *F*(1, 63) = 0.02, *p* = .888, $\eta_{P}^{2}$* < *.01. Again, we found no significant main effects of lag, *F*(1, 63) = 0.16, *p* = .690, $\eta_{P}^{2}$* < *.01, or category, *F*(1, 63)* < *0.01, *p* = .966, $\eta_{P}^{2}$* < *.01, on T1 accuracy in relation to the category of T2, and no interaction, *F*(1, 63) = 1.71, *p* = .196, $\eta_{P}^{2}$ = .03.

#### Additional analysis

We used signal detection theory to calculate sensitivity, *d’*, on yes or no responses on the pooled T2*|*T1 data from both experiments, excluding “I do not remember” responses (2%). For Lag 4, we got *d’* = 0.87 for animals and *d’* = 0.49 for artifacts. For Lag 7, we got *d’* = 1.44 for animals and *d’* = 1.0 for artifacts. Next, we calculated *d’* values for each participant, while replacing cells with 0 responses with 0.5. An ANOVA on lag (4,7) and category (animal, artifact) showed significant main effects of lag, *F*(1, 125) = 43.86, *p < *.001, $\eta_{P}^{2}$ = .26, and category, *F*(1, 125) = 44.67, *p < *.001, $\eta_{P}^{2}$ = .26. The interaction was not significant, *F*(1, 125) = 0.02, *p* = .881, $\eta_{P}^{2}$* < *.01.

### Discussion

Again, we were able to reveal an effect of lag and category but failed to observe an interaction with lag or a reduction of the advantage at the later lag. Hence, it seems that the response method used in Experiment 1 cannot account for the advantage for animals in the task. Also, the advantage for animals remains after increasing the frequency of animal distractors from 0% to 25%, in fact matching it to the frequency at which they were targets.

The results do however provide more evidence for an advantage for animals in this task, as it was replicated with a set of line drawings, whose low-level features are quite dissimilar from the sets of photographs used in previous experiments. This potentially rules out that the animacy effect could have been the result of some specific (possibly low-level) characteristics that are available in photographs (e.g., color or texture). Moreover, it suggests that a potential higher degree of ecological validity given by photographs is not so relevant for obtaining an advantage for animals in this task.

It is also interesting to note that the animal advantage was replicated regardless of the response mode (single probe instead of choice from a visual array). The visual array method may make guessing correctly less likely (given the multiple choices), but this aspect does not seem to be determinant for an advantage for animals. In fact, all three different stimuli sets tested so far have revealed a similar advantage for animals, despite the different manipulations for balancing them on potential nuisance variables.

## Experiment 3

A key aspect of the animate monitoring hypothesis is that animals should capture attention automatically. Several studies have shown attentional capture of biologically and emotionally relevant images as indexed by the induction of an attentional blink (Arnell et al., 2007; Ciesielski et al., 2010; Most et al., 2005; Neimeijer et al., 2013).Typically, in these studies, an image otherwise irrelevant to the task but thought to automatically capture attention is presented shortly before a neutral target image in an RSVP sequence; with the result that, the target is more difficult to report. Thus, in this experiment, we presented animals or artifacts as critical distractors before target images with the expectation that animal distractors would spontaneously capture attention and thereby induce attentional blinks, which would manifest in less successful target reporting.

One concern about Experiment 2 is that, by introducing multiple animal distractors, one might have induced a large amount of attentional blinks due to their ability to automatically capture attentional resources (New et al., 2007). Thus, the next experiment could also help resolve such a concern.

Specifically, in this experiment, we used either animals or artifacts as critical distractors and targets at Lag 2 or Lag 4. The prediction was that the automatic capture of attention by animals would induce an attention blink, therefore making any target presented shortly thereafter difficult to detect and report. In addition, we predicted that targets presented at Lag 2 would be more difficult to detect than those presented at Lag 4 (as is typically observed in attentional blink tasks).

A review of the literature suggests that we should expect at least a 10% decrease in reporting accuracy for targets at Lag 2 and 5% at Lag 4 after critical distractors as compared with neutral distractors. Power analysis revealed that we would need at least 12 participants to have 80% power to detect such a difference. However, to encompass an even smaller decrease of about 5% and 2.5% for Lags 2 and 4, we aimed to test approximately 45 participants.

### Methods

#### Participants

We recruited 50 participants for Experiment 3A (18 females) with a mean age of 33.8 years (range: 19–67 years, *SD*: 10.6 years) and 52 participants for Experiment 3B (16 females) with a mean age of 32.8 years (range: 18–56 years, *SD*: 9.7 years). All were recruited in the same way as the previous experiments.

#### Apparatus

Identical to the previous experiments.

#### Stimuli

Stimuli for Experiment 3A were identical to the stimuli in Experiment 2A (photographs). Stimuli for Experiment 3B were identical to the stimuli in Experiment 2B (line drawings).

#### Procedure

**Figure 8.**
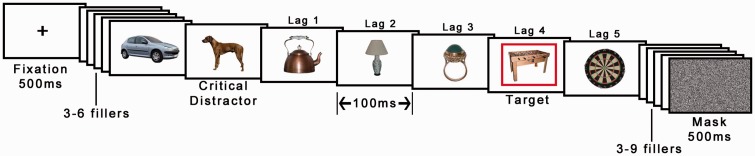
Example of a sequence in Experiment 3A. Only one object was presented within a red frame per trial. In this example, the target is presented at Lag 4 after the critical distractor animal (three intervening distractors). Animals appeared as critical distractors in 50% of trials. In this example, the expectation was that the dog should spontaneously recruit attention and thereby make the target at Lag 4 more difficult to report. Object images © 2012 Moreno-Martínez, Montoro, PLoS One, 7(5), p. e37528.

This was unchanged from Experiment 2 except that only one target was used and there were no animal distractors, except for trials in which animals acted as critical distractors (see Figure 8). The critical distractors were identical to those used as T2 targets in Experiment 2, the targets were randomly selected artifacts (previously used distractor objects).

### Results

#### Experiment 3A

**Figure 9.**
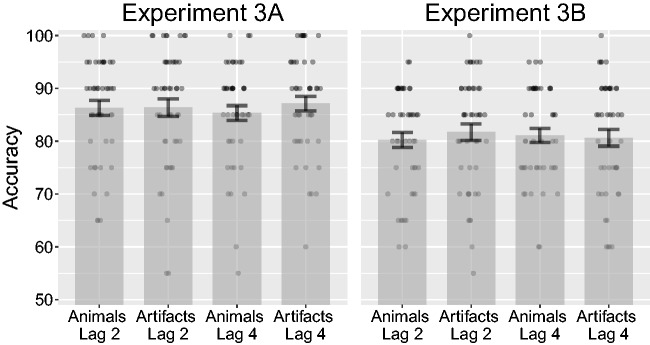
Combined bar and scatter plots on target accuracy from Experiments 3A and 3B. Error bars show standard error and the superimposed scatterplots show mean values from each participant. Experiment 3A used the same stimuli as in 2A (photographs), while Experiment 3B used the same stimuli as 2B (line drawings). Lags 2 and 4 correspond to 200 and 400 ms after onset of the critical distractor.

Before conducting the analysis, we removed three participants for having mean accuracy lower than 60% (6% of all trials). An ANOVA on target accuracy over lag (2, 4) and category (animals, artifacts) did not indicate a significant main effect of lag, *F*(1, 46) = 0.01, *p* = .916, $\eta_{P}^{2}$* < *.01, or category, *F*(1, 46) = 0.71, *p* = .405, $\eta_{P}^{2}$ = .02. The interaction was not significant, *F*(1, 46) = 0.69, *p* = .410, $\eta_{P}^{2}$ = .01 (see Figure 9). Targets at Lag 2 after animal distractors had a mean accuracy of 86.3% (*SD* = 9.69%), while targets at Lag 2 after artifact distractors had a mean accuracy of 86.4% (*SD* = 11.5%). Similarly, targets at Lag 4 after animal distractors had a mean accuracy of 85.3% (*SD* = 9.75%), while targets at Lag 4 after artifact distractors had a mean accuracy of 87.1% (*SD* = 9.54%).

#### Experiment 3B

Before conducting the analysis, we removed six participants for having mean accuracy lower than 60% (11% of all trials). An ANOVA on target accuracy over lag (2, 4) and category (animals, artifacts) did not indicate a significant main effect of lag, *F*(1, 45) = 0.01, *p* = .938, $\eta_{P}^{2}$* < *.01, or category, *F*(1, 45) = 0.18, *p* = .675, $\eta_{P}^{2}$* < *.01. The interaction was not significant, *F*(1, 45) = 0.58, *p* = .451, $\eta_{P}^{2}$ = .1 (see Figure 9). Targets at Lag 2 after animal distractors had a mean accuracy of 80.2% (*SD* = 9.72%), while targets at Lag 2 after artifact distractors had a mean accuracy of 81.7% (*SD* = 10.7%). Similarly, targets at Lag 4 after animal distractors had a mean accuracy of 81.1% (*SD* = 9%), while targets at Lag 4 after artifact distractors had a mean accuracy of 80.7% (*SD* = 10.9%).

### Discussion

We found no support in this experiment for the hypothesis that animals spontaneously and automatically capture attention and thereby make the reporting of any targets presented shortly thereafter more difficult. Neither was there any indication of an attentional blink or effect of type of critical distractors. These results are consistent with the results from the previous two experiments and strengthen the conclusion that animal images neither reduce nor induce attentional blinks. One concern about these experiments could be that participants were instructed to detect a red frame containing the target object and thereby might have effectively ignored anything not within a red frame. However, this task demand should not be of concern for the animate monitoring hypothesis (New et al., 2007), which states that animals are automatically and autonomously prioritized regardless of task demands or instructions.

## Experiment 4

Previous research has only described an advantage for animals over artifacts in reporting the second target. Our results suggest a general advantage in animal target reporting that is not specific to the attentional blink. In other words, the simple occurrence of an animal as a single target (i.e., T1 without T2) should show an advantage as well. Thus, in this experiment, we had only one target per trial, which could be either an animal or an artifact. We expected that animals would be reported more accurately than artifacts, supporting the idea that such an advantage is not specific to the attentional blink.

### Methods

#### Participants

We recruited 54 participants for Experiment 4A (13 females) with a mean age of 33.4 years (range: 19–55 years, *SD*: 8.94 years) and 49 participants for Experiment 4B (18 females) with a mean age of 33.8 years (range: 19–51 years, *SD*: 9.08 years). All were recruited in the same way as the previous experiments.

#### Apparatus

Identical to the previous experiments.

#### Stimuli

Stimuli for Experiment 4A were identical to the stimuli in Experiment 2A. Stimuli for Experiment 4B were identical to the stimuli in Experiment 2B.

#### Procedure

**Figure 10.**
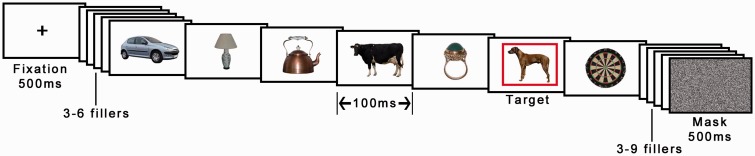
Illustration of the procedure in Experiment 4. Only one object was designated as a target by being shown in a red frame per trial and this could be either an animal or an artifact. Object images © 2012 Moreno-Martínez, Montoro, PLoS One, 7(5), p. e37528.

The procedure was identical to Experiment 2, except that we used one target only, which could be equally likely either animal or artifact (see Figure 10).

### Results

#### Experiment 4A

**Figure 11.**
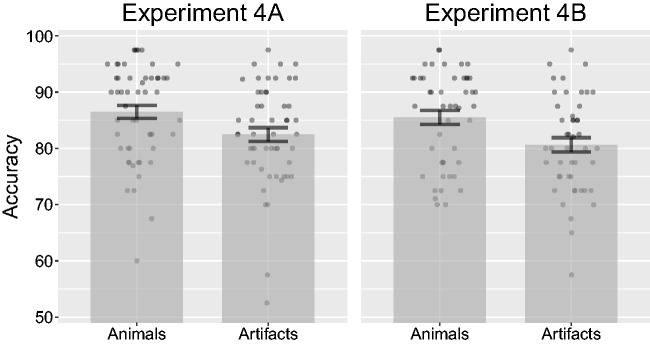
Combined bar and scatter plots on target accuracy from Experiments 4A and 4B. Error bars show standard error and the superimposed scatterplots show mean values from each participant. Experiment 4A used the same stimuli as in 2A (photographs), while Experiment 4B used the same stimuli as 2B (line drawings).

Before conducting the analysis, we removed one participant for having mean accuracy lower than 60% (1.9% of all trials). An ANOVA on target accuracy revealed a significant effect of category, *F*(1, 52) = 10.03, *p* = .003, $\eta_{P}^{2}$ = .16 (see Figure 11). Animal targets had a mean accuracy of 86.5% (*SD* = 8.57%), while artifact targets had a mean accuracy of 82.5% (*SD* = 8.89%).

#### Experiment 4B

Before conducting the analysis, we removed two participants for having mean accuracy lower than 60% (4.1% of all trials). Again, an ANOVA on target accuracy revealed a significant effect of category, *F*(1, 46) = 15.81, *p < *.001, $\eta_{P}^{2}$ = .26 (see Figure 11). Animal targets had a mean accuracy of 85.5% (*SD* = 8.45%), while artifact targets had a mean accuracy of 80.6% (*SD* = 8.6%).

### Additional Analysis

To investigate if advantages for animals in this experiment were significantly different from the advantages in Experiment 2, we subtracted T2*|*T1 accuracy for artifact targets from animal targets at both Lags 4 and 7 in Experiments 2A and 2B. Advantages were calculated in the same manner for Experiments 4A and 4B. An ANOVA on advantages between Experiments 2A Lag 4 and 4A showed no significant difference, *F*(1, 113) = 1.02, *p* = .314, $\eta_{P}^{2}$* < *.01. Similarly, an ANOVA between Experiments 2A Lag 7 and 4A also showed no significant difference, *F*(1, 113) = 1.96, *p* = .165, $\eta_{P}^{2}$ = .02. Next, an ANOVA between Experiments 2B Lag 4 and 4B showed no significant difference, *F*(1, 109) = 0.20, *p* = .653, $\eta_{P}^{2}$* < *.01. Finally, an ANOVA between Experiments 2B Lag 7 and 4B also showed no significant difference, *F*(1, 109) = 0.12, *p* = .735, $\eta_{P}^{2}$* < *.01.

### Discussion

We found support for the prediction that animals should be reported more accurately than artifacts when having to report only a single target. This result thus expands on the observation that animals are detected more accurately in this task to also include single targets. Moreover, these finding strongly suggest that the advantages we observed in the previous experiments were most likely not due to a reduction of the attentional blink but to a general perceptual speed factor that favors animals.

## Experiment 5

In Experiment 3, we observed that animals did not spontaneously induce attentional blinking, but in Experiments 1, 2, and 4, we observed robust advantages for second and single target animals. One possibility is that animal targets capture attention more intensely than artifact targets. If this is the case, then we should expect this to be measurable in a more pronounced attentional blink. Thus, in this experiment, we investigated whether animals presented as T1 would lead to more difficulty in reporting T2 images than when artifacts presented as T1. Similar to Experiment 3, we chose to use Lags 2 and 4 for the T2 images. In addition, the results from Experiments 1, 2, and 4 suggest that animal targets should be reported more accurately than artifact targets.

### Methods

#### Participants

For Experiment 5A, we recruited 53 participants (18 females) with a mean age of 32.2 years (range: 17–51 years, *SD*: 8.02 years). For Experiment 5B, we recruited 60 participants (12 females) with a mean age of 31.3 years (range: 18–55 years, *SD*: 8.75 years).

#### Apparatus

Identical to the previous experiments.

#### Stimuli

Stimuli for Experiment 5A were identical to the stimuli in Experiment 2A (photographs). Stimuli for Experiment 5B were identical to the stimuli in Experiment 2B (line drawings). In addition, we switched the sets of images used as T1 and T2 such that animals and artifacts became T1 images.

#### Procedure

**Figure 12.**
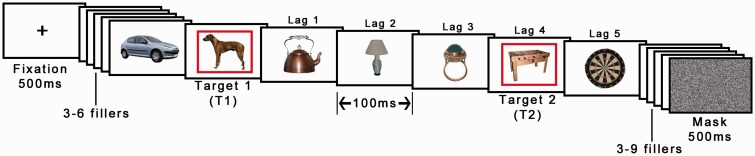
Illustration of the procedure in Experiment 5. Object images © 2012 Moreno-Martínez, Montoro, PLoS One, 7(5), p. e37528.

The procedure was identical to Experiment 2, except that we used Lags 2 and 4 for the second target (see Figure 12 for an example).

### Results

#### Experiment 5A

**Figure 13.**
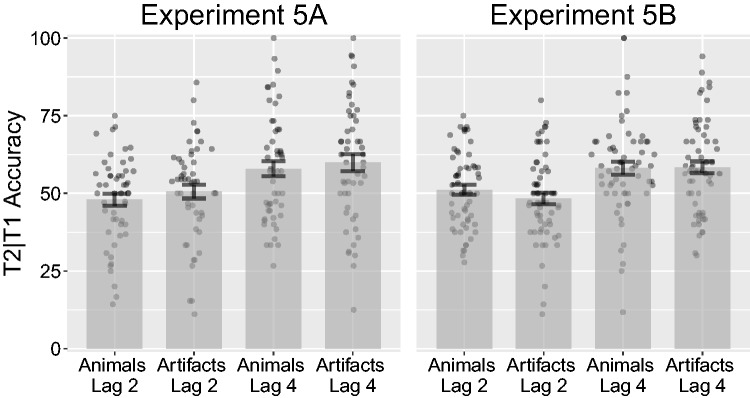
Combined bar and scatter plots on T2*|*T1 accuracy from Experiments 5A and 5B over T1 category and T2 lags. Error bars show standard error and the superimposed scatterplots show mean values from each participant. Experiment 5A used the same stimuli as in 2A (photographs), while Experiment 5B used the same stimuli as 2B (line drawings).

**Figure 14.**
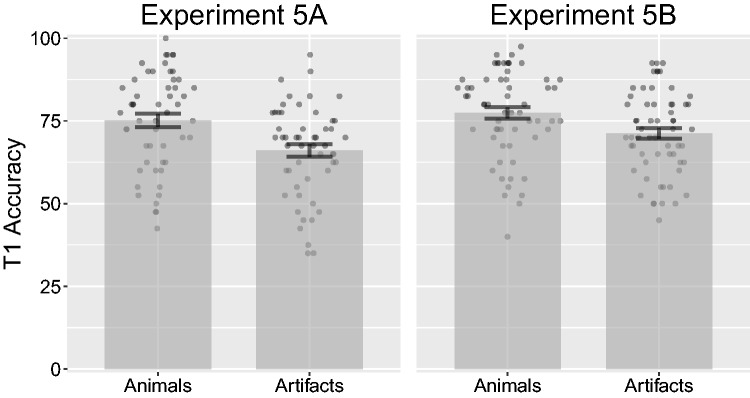
Combined bar and scatter plots on T1 accuracy from Experiments 5A and 5B. Error bars show standard error and the superimposed scatterplots shown mean values from each participant.

Before conducting the analysis, we removed two participants for having mean T1 accuracy lower than 40% (3.7% of all trials). An ANOVA on T2*|*T1 accuracy over T1 category (animal, artifact) and lag (2, 4) revealed a non-significant effect of T1 category *F*(1, 50) = 1.74, *p* = .193, $\eta_{P}^{2}$ = .03, and a significant effect of lag, *F*(1, 50) = 27.02, *p < *.001, $\eta_{P}^{2}$ = .35. The interaction was not significant, *F*(1, 50) = 0.04, *p* = .851, $\eta_{P}^{2}$* < *.01 (see Figure 13). Targets at Lag 2 after correctly reported animals had a mean accuracy of 48% (*SD* = 14.2%), while targets at Lag 2 after correctly reported artifacts had a mean accuracy of 50.5% (*SD* = 15.7%). At Lag 4, targets after correctly reported animals had a mean accuracy of 57.9% (*SD* = 17.6%), while targets after correctly reported artifacts had a mean accuracy of 59.9% (*SD* = 19.5%). Next, an ANOVA on T1 accuracy on category revealed a significant difference, *F*(1, 50) = 34.50, *p < *.001, $\eta_{P}^{2}$ = .41. T1 animals had a mean accuracy of 75.2% (*SD* = 14.7%), while artifacts had a mean accuracy of 66.1% (*SD* = 13.9%), for illustration, see Figure 14.

#### Experiment 5B

An ANOVA on T2*|*T1 accuracy over T1 category (animal, artifact) and lag (2, 4) revealed a non-significant effect of T1 category, *F*(1, 59) = 0.56, *p* = .457, $\eta_{P}^{2}$* < *.01, and a significant effect of lag, *F*(1, 59) = 22.02, *p < *.001, $\eta_{P}^{2}$ = .27. The interaction was not significant, *F*(1, 59) = 0.80, *p* = .376, $\eta_{P}^{2}$ = .01 (see Figure 13). Targets at Lag 2 after correctly reported animals had a mean accuracy of 51.1% (*SD* = 12.3%), while targets at Lag 2 after correctly reported artifacts had a mean accuracy of 48.3% (*SD* = 14.2%). At Lag 4, targets after correctly reported animals had a mean accuracy of 58.1% (*SD* = 15.8%), while targets after correctly reported artifacts had a mean accuracy of 58.3% (*SD* = 15%). Next, an ANOVA on T1 accuracy on category revealed a significant difference, *F*(1, 59) = 27.30, *p < *.001, $\eta_{P}^{2}$ = .32. T1 animals had a mean accuracy of 77.4% (*SD* = 13.3%), while artifacts had a mean accuracy of 71.2% (*SD* = 12.6%), for illustration, see Figure 14.

### Additional Analysis

To investigate if T1 advantages for animals in this experiment were significantly different from the advantages in Experiment 2, we subtracted T2*|*T1 accuracy for artifact targets from animal targets at both Lags 4 and 7 in Experiments 2A and 2B. Advantages were calculated in the same manner based on T1 accuracies for Experiments 5A and 5B. An ANOVA on advantages between Experiments 2A Lag 4 and 5A showed no significant difference, *F*(1, 113) = 0.28, *p* = .598, $\eta_{P}^{2}$* < *.01. Similarly, an ANOVA between Experiments 2A Lag 7 and 5A also showed no significant difference, *F*(1, 113) = 0.32, *p* = .570, $\eta_{P}^{2}$* < *.01. Next, an ANOVA between Experiments 2B Lag 4 and 5B showed no significant difference, *F*(1, 122)* < *0.01, *p* = .967, $\eta_{P}^{2}$* < *.01. Finally, an ANOVA between Experiments 2B Lag 7 and 5B also showed no significant difference, *F*(1, 122) = 0.01, *p* = .914, $\eta_{P}^{2}$* < *.01.

### Discussion

The findings did not support the prediction that animal T1 images should lead to more difficulty in reporting T2 images, as T2*|*T1 accuracy were not significantly different between categories at either lags.

However, the observation that animal T1 images were reported more accurately than artifact T1 images is consistent with the previous experiments. This further strengthens the conclusion that animals can be reported more accurately than artifacts, irrespective of their temporal position. Similar suggestions were provided by the additional analysis of advantages between the present experiment and Experiment 2. This also indicated that advantages in this experiment were not significantly different from advantages observed when the same targets were presented as second targets within and outside the attentional blink window. Balas and Momsen (2014) used a similar design by presenting animals and plants as T1. In line with our results, they found that animals were reported more accurately than plants. However, their observations of differential T2 accuracies depending on the type of T1 image were not observed in the present experiment. One set of reasons for this could be that they used considerably faster image presentations (32 ms) as well as a different set of instructions, namely to specifically detect plants and water (Group 1) or animals and water (Group 2). Another aspect could be that they used full-frame color photographs with backgrounds; thus, low-level aspects correlated with the types of images used or the ease at which the different objects can be segregated from their backgrounds could have played a role. Alternately, it could be that animal and artifact T1 images lead to similar T2 accuracies, but that T1 images of plants lead to differential T2 accuracies from both animals and artifacts.

## General Discussion

All of the present experiments with animal and artifact targets revealed reporting advantages for animal targets, and with three different sets of images, regardless if they were shown inside or outside of the range of the typical attentional blink window. Thus, they extend the previous observation of an advantage for animals in T2 reporting (Guerrero & Calvillo, 2016) and provide evidence that such an animal advantage did not stem from a reduction of the attentional blink per se. In addition, we failed to observe any spontaneous inductions of attentional blinks when displaying single animal images as distractors shortly before targets. Finally, we also observed an advantage for animals over artifacts when they appeared as the first of two targets or as single targets, which suggest that the advantage is general to reporting targets in this task and not specific to second target reports or within the typical attentional blink’s window. Thus, in contrast to previous work, we found that animals need not be presented as the second target or be part of a dual reporting task to show a reporting advantage.

While the present study failed to verify an attentional prioritization of animals, as predicted by the animate monitoring hypothesis (New et al., 2007), it does provide strong evidence for a general reporting advantage for animals with RSVP tasks. As the combined results suggest that animals do not induce or reduce attentional blinking but are reported more accurately whether presented within or outside the attentional blink window, this animal bias is most likely related to later, post-attentive, processing stages.

Nevertheless, a higher accuracy for animal targets is in line with the previous results of Guerrero and Calvillo (2016), but the effect does not appear to be related to a verbal response method since it was found in this study also when we used visual arrays and single probes. Moreover, the effect was observed with three different stimuli sets and despite varying the ratios of animal or artifact distractors. One possibility is that animals are either more efficiently encoded at the perceptual stage or better, more robustly, retained in short-term memory after encoding. Indeed, there are already several studies indicating memory advantages for animals (Bonin, Gelin, & Bugaiska, 2014; Nairne, VanArsdall, & Cogdill, 2017; Nairne, VanArsdall, Pandeirada, Cogdill, & LeBreton, 2013; VanArsdall, Nairne, Pandeirada, & Blunt, 2013).

Further research could investigate the earlier aspects by testing for the presence of an animal advantage at variable presentation durations. If animals require less information uptake to be encoded sufficiently, then the advantage over artifacts should decline at longer presentation durations and increase at shorter presentation durations. Interestingly, Laws and Neve (1999) showed higher naming accuracy for animals over artifacts with 20 ms presentations (but see Låg, 2005). Whether the correct account implies more efficient encoding or stronger retention, these processes does not need to reflect an attentional bias as well. In fact, we failed to observe any inductions or reductions of attentional blinking for animal targets, which has been shown for other categories of stimuli involved in attentional biases (Anderson, 2005; de Oca, Villa, Cervantes, & Welbourne, 2012; Fox et al., 2005; Milders et al., 2006; Ogawa & Suzuki, 2004; Stein et al., 2010).

As the aim of this study was to investigate if images of animals can induce or reduce attentional blinking, we did not directly aim to investigate the source of a potential general advantage for animals. Thus, it is plausible that a host of uncontrolled variables could have influenced our results. In Experiment 1B, we did control for several low-level visual characteristics, while in Experiment 2B, we controlled for familiarity and visual complexity. As the stimuli sets were visually quite different, it appears less likely that low-level visual features, other than the shape or outline of the objects, contributed to the effect. A more plausible account thus may stem from consideration about the global shape of biological objects (tending to be “curved” instead of “carpentered”; Laeng & Caviness, 2001; Long, Störmer, & Alvarez, 2017), semantic factors such as age of acquisition, imageability, within-category similarity, and so forth, or a general advantage in memory (Nairne et al., 2017).

Interestingly and contrary to this suggestion, studies on memory advantages for animate words and images have suggested that the advantage could stem from an attentional bias (Bonin et al., 2014; VanArsdall et al., 2013). The reasoning being that if animals recruit more attention, their encoding and retention will be enhanced as well.Following these suggestions, a functional magnetic resonance imaging study (Xiao, Dong, Chen, & Xue, 2016) on memory for words for living and nonliving items examined whether an attentional or a semantic account was more likely to account for the pattern of results. It was found that activity in the dorsal attention network could not predict memory performance or mediate the animacy effect. However, there was evidence in favor of an overlapping-semantic-features account for the animal memory advantage. This view is also in line with what was proposed by Laws and Neve (1999).

Future studies could try to disentangle the source of the advantage for animal images in this task with even more controlled efforts. If it turns out that animals are truly more efficiently encoded, despite not detected faster in a change detection task (Hagen & Laeng, 2016), or unable to influence attentional blinks, then an attentional explanation for this efficiency would not seem necessary. Other factors such as the ease of recovering identity from global shapes or uncontrolled variables could represent more plausible explanations of these patterns of results. An attentional bias that is only measurable as more efficient encoding or retention and not sensitive to typical attention tasks would appear quite peculiar and, at any rate, would fail to support the idea of an automatic bias or prioritization in attention. It could also be relevant to use nonanimal natural objects to assess whether the advantage is general to the broader class of natural objects. It could perhaps also be interesting to display the objects upside down (Livesey & Harris, 2011) in an attempt to control for the ease at which objects are recognized. The present study focused on animal images rather than animate images in general, since we did not include images of humans (or robots). Thus, future studies could investigate if images of humans reduce the attentional blink as well, although there might already be some support from facial stimuli (Landau & Bentin, 2008). Correspondingly, it could be interesting to investigate if the presence of facial features on animals has any impact on their advantage in this task.

In Experiment 3, we could have attempted to tune participants to a deeper level of processing per image before designating them as targets or distractors by using a task where targets are rotated 90° coupled with an instruction to detect images at 90° angles (e.g., Ciesielski et al., 2010). However, this seems like an unnecessary requirement with regard to the supposedly automatic and effortless prioritization of animals (New et al., 2007).

It is still possible that we have made a Type II error in failing to reject the null hypothesis that animals are reported more accurately regardless of being presented within or outside the typical attentional blink window. However, this seems unlikely as we replicated a similar pattern of results in five experiments and should have had enough power to detect even smaller differences than those suggested by the current literature. Similarly, we could have made a Type I error in not rejecting the hypothesis that animals are generally reported more accurately than artifacts in the RSVP task. Independent observations with different approaches should help clarify the robustness of these observations.

Finally, a caveat that is common to all endeavors to investigate the perception and attention to animals is that the animal stimuli, in the laboratory context, do not actually represent authentic survival value. That is, participants understand that the presented stimuli are not real animals and therefore it may not be relevant to prioritize such symbolic, fictional, stimuli. If such a higher order control on attention and perception is really possible, then laboratory- or computer-based studies of evolutionary mechanism like the animate monitoring hypothesis may be doomed to fail. However, it is important to note that studies showing images of emotional faces or other biologically relevant stimuli have shown clear advantages in the laboratory with symbolic representations; therefore, it seems reasonable to conclude that the available evidence is not supportive of the animate monitoring hypothesis.
